# Liquid Crystals Based on the *N*-Phenylpyridinium Cation—Mesomorphism and the Effect of the Anion

**DOI:** 10.3390/molecules26092653

**Published:** 2021-05-01

**Authors:** Jordan D. Herod, Duncan W. Bruce

**Affiliations:** Department of Chemistry, University of York, Heslington, York YO10 5DD, UK; jordan.herod94@gmail.com

**Keywords:** liquid crystal, ionic, polycatenar, ionic liquid

## Abstract

Families of symmetric, ionic, tetracatenar mesogens are described based on a rigid, *N*-phenylpyridinium core, prepared as their triflimide, octyl sulfate and dodecyl sulfate salts for a range of terminal chain lengths. The mesomorphism of the individual series is described before a comparison is drawn between the different families and then more broadly with (i) neutral tetracatenar materials and (ii) related bis(3,4-dialkoxystilbazole)silver(I) salts. For the octyl and dodecyl sulfates and the related triflates reported earlier, a SmA phase is seen at shorter chain lengths, giving way to a Col_h_ phase as the terminal chain lengthens. For the alkyl sulfate salts, an intermediate cubic phase is also seen and the terminal chain length required to cause the change from lamellar to columnar mesophase depends on the anion. Furthermore, there is an unexpected and sometime very large mesophase stabilisation seen on entering the columnar phase. All of the triflimide salts show a rectangular columnar (ribbon) phase.

## 1. Introduction

The first formally ionic liquid crystals can be traced back to the work of Heinz in the mid-19th century with magnesium tetradecanoate [1], followed (inevitably) by work on other metal carboxylates from Vorländer [2]. Through systematic studies of carboxylate salts starting in the late 1950s [3] and of alkylammonium halometallates in the 1970s [4], ionic liquid crystals eventually collided with the burgeoning field of ionic liquids, along with the first reports of mesomorphic imidazolium salts in the mid-1990s [5]. The field has now expanded significantly and ionic liquid crystals are a unique class of materials as they combine the properties inherent to ionic liquids with the long-range anisotropic order of liquid crystals.

Of contemporary interest in liquid crystal science are the observations that come from combining, in the same molecular system, functionalities that drive self-organisation by different or complementary mechanisms. For example, the self-organisation of polycatenar materials is influenced strongly when both hydrocarbon and semi-perfluorocarbon chains are incorporated into the same mesogen [6]. Equally, there is interest in multifunctional liquid crystals and, for example, Kato et al. [7]. demonstrated that ionic liquid crystals forming columnar mesophases can show temperature-dependent ion conductivity. Similarly, liquid-crystalline viologens remain a topic of interest due to their redox behaviour that can be combined with liquid-crystalline self-assembly to produce redox materials with anisotropic properties [8,9,10,11,12,13,14].

Recently, we reported the synthesis and mesomorphism of some polycatenar ionic mesogens based on *N*-phenylpyridinium triflates [15], noting related studies on calamitic *N*-phenylpyridinium salts [8,16] and some wedge-shaped *N*-phenylpyridium salts [17] by Lai et al. The study was prompted both by a wish to compare truly ionic polycatenar mesogens with the polycatenar stilbazole silver(I) salts that we had reported over several years, and also to investigate how the different driving forces for mesophase formation between ionic mesogens (electrostatics) and polycatenar systems (chain/core interfaces and volumes) may be accommodated simultaneously. The study has since been extended to salts with different anions, the results of which are now reported.

## 2. Results

### 2.1. Synthesis

**Figure 1:**
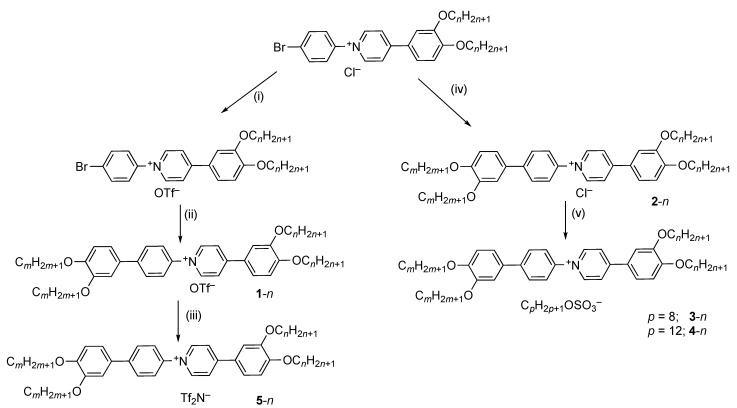
Preparation of the target salts; in all cases *n* = *m*. (**i**) AgOTf, DMF, 70 °C; (**ii**) 3,4-dialkoxybenzene boronic acid pinacol ester, THF/H_2_O (1:1), Na_2_CO_3_, N_2_, [Pd_3_(OAc)_6_], SPhos, 65 °C; (**iii**) LiTf_2_N/MeOH/Δ; (**iv**) 3,4-dialkoxybenzene boronic acid pinacol ester, THF/H_2_O/EtOH (3:3:1), Na_2_CO_3_, [Pd_3_(OAc)_6_], SPhos, N_2_, 65 °C; (**v**) NaO_3_SOC*_p_*H_2*p*+1_/MeOH/Δ (*p* = 8 or 12).

The synthesis of the *N*-phenylpyridinium triflates was as reported previously, but Figure 1 shows modifications that allowed access to the related triflimides and alkyl sulfates. Thus, heating under reflux a LiTf_2_N-saturated methanol solution of a triflate (**1**-*n*) for three hours followed by dropwise addition of water to the cooled solution led to the precipitation of the triflimide salts (**5**-*n*) in 69%–83% yield (dependent on aliphatic chain length) following extensive washing and drying. However, direct metathesis from triflate was not effective for alkyl sulfates as ^19^F NMR spectroscopy showed that the exchange was not complete. Therefore, a different route was devised in which the tetracatenar chloride salt (**2**-*n*) was first obtained using a Suzuki-Miyaura protocol from *N*-(4-iodophenyl)-4-(3,4-dialkoxyphenyl)pyridinium chloride (**2**-*n*). These chloride salts were not very soluble and as such they were difficult to purify (satisfactory elemental analyses were never obtained). However, when stirred with a saturated solution of the appropriate sodium alkyl sulfate in hot methanol (shorter-chain cations) or hot ethanol/methanol (longer-chain cations) and precipitating by dropwise addition of water, they led smoothly to the octyl sulfate salts **3**-*n* in yields of 66–82% and dodecyl sulfate salts **4**-*n* in yields of 65%%–87% following extensive washing and drying. Analytical data for all new salts are found in Tables S1–S3.

### 2.2. Mesomorphism of the Octyl Sulfate Salts (***3***-n)

**Figure 2:**
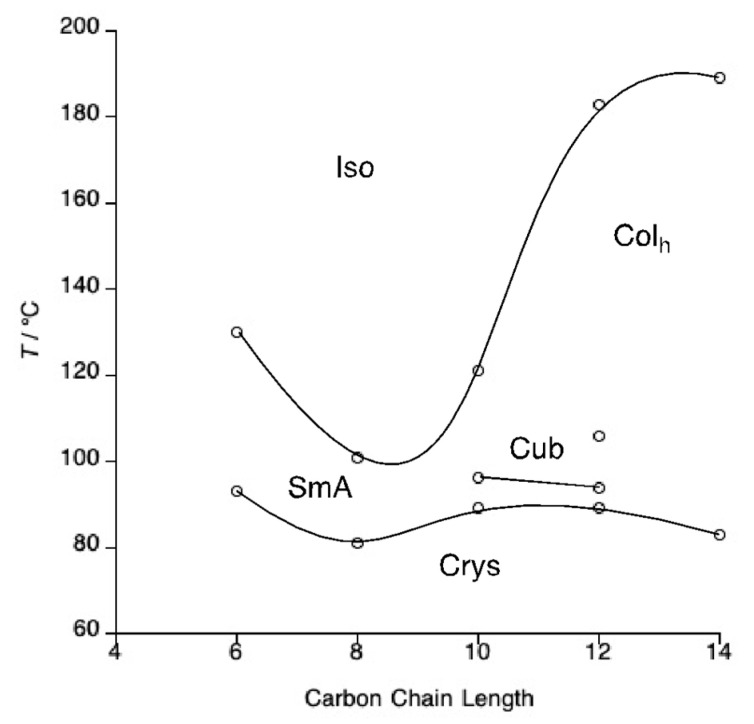
Phase diagram of the tetracatenar *N*-phenylpyridinium octyl sulfate salts, **3**-*n*.

**Figure 3:**
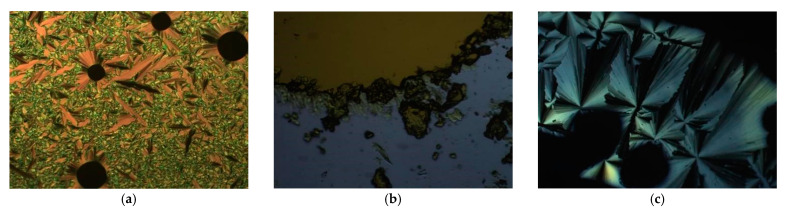
Optical textures of (**a**) the SmA phase formed by compound **3**-6 at 116 °C, (**b**) the cubic phase formed by **3**-10 with polarisers slightly uncrossed at 105 °C, (**c**) the Col_h_ phase formed by compound **3**-12 at 154 °C.

The mesomorphism of these and the other salts under study was investigated by first examining the phase behaviour using polarised optical microscopy. Thus, the phase diagram of the octyl sulfate salts, **3**-*n*, is presented in Figure 2, their transition temperatures are collected in Table S4 and optical textures are found in Figure 3. In common with the triflate salts **1**-*n*, the shorter-chain homologues melted (**3**-6 at 93 °C and **3**-8 at 81 °C) to give a SmA phase, readily identified by its optical texture that showed a characteristic focal conic fan texture, which persisted to clearing points of 130 and 101 °C, respectively. Perhaps unsurprisingly given the greater volume occupied by the anion and its flexibility, the melting points of these octyl sulfate salts was a little lower than those of the corresponding triflates. When the cation terminal chain reached ten carbons, the range of the SmA phase reduced to 7 °C and a cubic phase was seen above it which persisted to the clearing point at 121 °C and which was characterised by its isotropic nature and elevated viscosity compared to the SmA phase. SmA and cubic phases were seen also for **3**-12 with ranges of 5 and 12 °C, respectively, but then the cubic phase gave way to a columnar phase at 106 °C that persisted to the clearing point at 183 °C. At **3**-14, neither the SmA nor the cubic phase was observed, and the salt simply melted directly to the columnar phase at 83 °C, persisting through to clearing at 189 °C. Interestingly, none of the salts crystallised on cooling; rather they formed a glassy mesophase, with the glass transition being observed by DSC (Figure S6).

Pseudo focal-conic textures with spine-like defects were observed for the columnar phase formed by compounds **3**-14 and **3**-12 (Figure 3c shows the texture for **3**-12), suggesting strongly that the phase has hexagonal symmetry.

The mesophases were investigated further by small-angle X-ray scattering (SAXS) and the data are collected in Table S5. The SmA phases of **3**-6 and **3**-8 showed characteristic (001) reflection (with additional (002) for **3**-6) with accompanying diffuse, wide-angle reflection, and the observed *d*-spacings (26.4 and 28.7 Å for **3**-6 and **3**-8, respectively) were shorter than the length of the cation (e.g., 38.9 Å for **3**-8 as determined from the X-ray single crystal structure of **1**-8 [15]). It was only possible to stabilise **3**-10 in its cubic phase and here two reflections were observed, a strong one corresponding to a spacing of 30.5 Å and a much weaker shoulder at a spacing of 26.6 Å. It has not been possible to index these with any confidence and the absence of higher-order reflections precludes the identification of a space group.

SAXS data for **3**-14 showed two reflections at spacings of 33.1 and 19.1 Å, which indexed as the (10) and (11) reflections of a hexagonal plane group and were consistent with the predictions from optical microscopy. Unfortunately, the (11) reflection was not observed for **3**-12, but identification of the phase as having hexagonal symmetry was confirmed, as the columnar phases of **3**-12 and **3**-14 were found to be co-miscible.

### 2.3. Mesomorphism of the Dodecyl Sulfate Salts (***4***-n)

**Figure 4:**
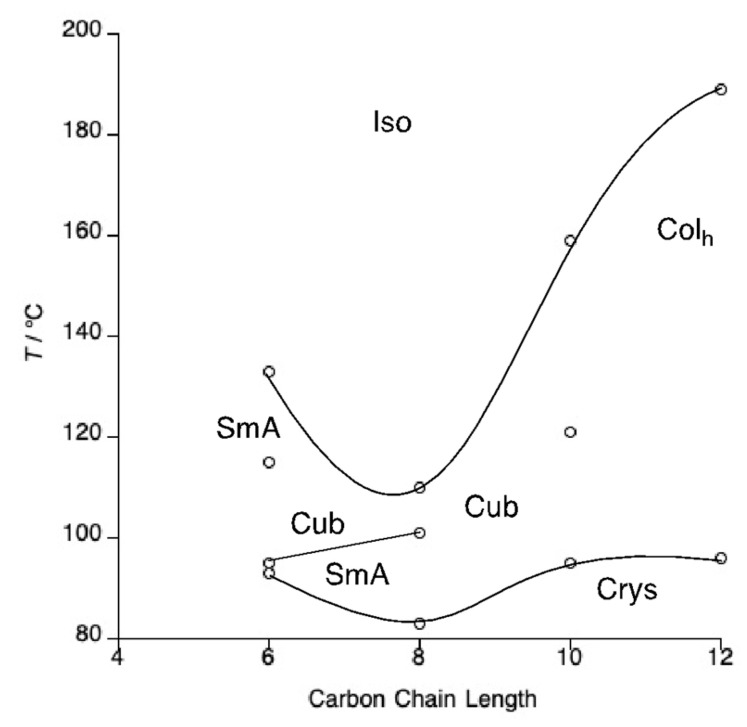
Phase diagram of the tetracatenar *N*-phenylpyridinium dodecyl-sulfates, **4**-*n*.

**Figure 5:**
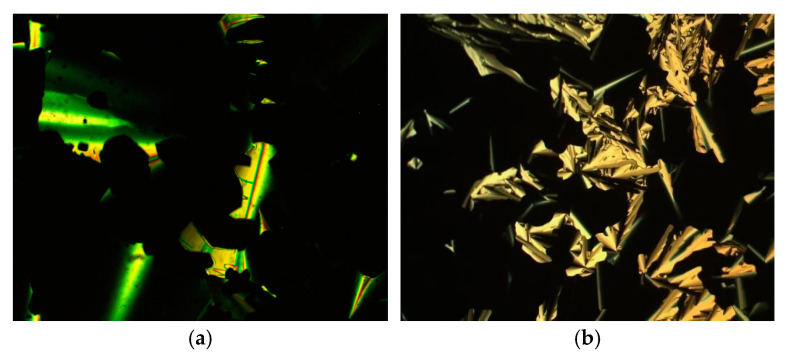
(**a**) The cubic phase growing in from Col_h_ phase of compound **4**-10 at 108 °C and (**b**) the Col_h_ phase formed by compound **4**-12 at 172 °C.

The phase diagram of the corresponding dodecyl sulfate salts, **4**-*n*, is presented in Figure 4 with the data collected in Table S6. The overall pattern of behaviour mirrors that of the octyl sulfate salts, namely a progression from a lamellar phase to a columnar phase with increasing terminal chain length, but there are aspects of detail that are different. Thus, on heating **4**-6, the solid melts at 93 °C to a SmA phase which gives way almost immediately (95 °C) to a cubic phase which persists for 20 °C when a SmA phase is reformed that eventually clears at 133 °C. This unusual mesomorphism was reproducible independent of heating rate, although it is interesting that the cubic mesophase was never observed on cooling from the isotropic liquid and a SmA glass eventually forms on cooling. Salt **4**-8 also melts to a SmA phase (83 °C), but this time more conventional behaviour was observed and a cubic phase was observed from 101 to 110 °C. The mesomorphism of **4**-10 and **4**-12 were dominated by the formation of a Col_h_ phase, with **4**-10 also showing a cubic phase and neither showing a SmA phase. The clearing points of the Col_h_ phases were very much higher than those of the cubic or SmA phases. Phases were once more identified by optical microscopy (Figure 5) and once more, pseudo focal-conic textures with spine defects were observed on cooling compounds **4**-10 and **4**-12 from the isotropic liquid to identify the hexagonal phase as columnar. Figure 5a shows the square edges of the cubic phase growing in from the Col_h_ phase in **4**-10.

**Figure 6:**
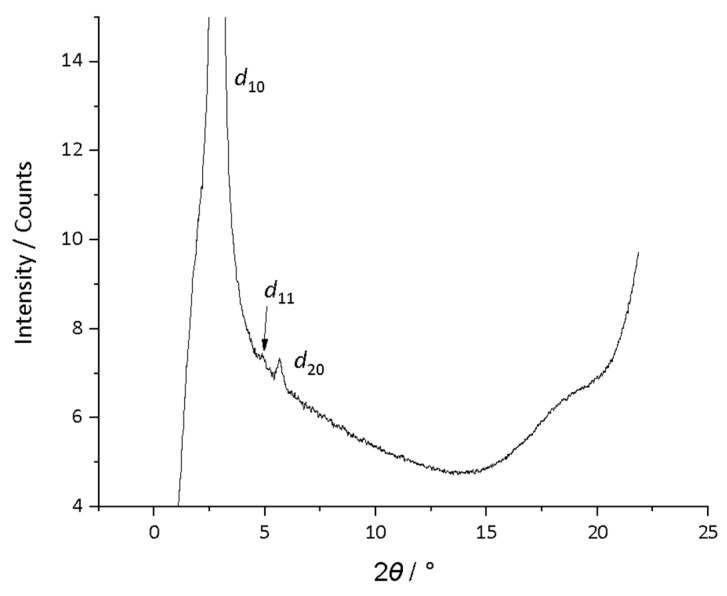
Diffraction pattern of the Col_h_ phase formed by compound **4**-10 at 105 °C on cooling.

X-ray diffraction data for series **4**-*n* are collected in Table S7. A single reflection was observed in the diffraction patterns of compounds **4**-6 and **4**-8 that is consistent with the formation of a SmA phase. The observed spacings of 26.8 and 28.8 Å, respectively, are all but identical to those of the corresponding octyl-sulfate salts and show that the anion does not have an effect on the layer spacing. Compounds **4**-10 and **4**-12 both showed the (10) and (20) reflections of the hexagonal phase with the (11) reflection also being observed for **4**-10 (Figure 6). For the cubic phase of both **4**-8 and **4**-10, two small-angle reflections were found but once more there were insufficient data to begin to assign the space group.

On comparing the phase diagrams of the octyl sulfate and dodecyl sulfate materials (series **3**-*n* and **4**-*n*, respectively), it becomes apparent that the transition to Col_h_ mesomorphism takes place at a shorter alkoxy chain length for the dodecyl sulfate materials. The melting points remain broadly similar across both series so that the additional methylene groups of the dodecyl sulfate anion are not sufficient to destabilise the crystal phase further. In addition, compounds **3**-*n* and **4**-*n* display a similar trend in their clearing points in that the SmA phases formed at short terminal chain lengths are steadily destabilised on increasing terminal chain length; then, at the transition to Col_h_ mesomorphism, the clearing point increases dramatically.

### 2.4. Mesomorphism of the Triflimide Salts (***5***-n)

For some time, there was a perception that triflimide would tend to suppress liquid crystal phase formation in ionic systems and perhaps the origin of this thought is that where the cation is small, for example in imidazolium salts, the size of the triflimide anion is destabilising as it will compromise structural anisotropy. However, where the anion is larger, for example with viologens [8,9,10,11,12,13,14] or 3-phenyl-1,2,4-triazines [18,19], then triflimide will indeed support liquid crystal behaviour. Therefore, the triflimide salts of these *N*-phenylpyridium cations were prepared and their mesomorphism is now described.

**Figure 7:**
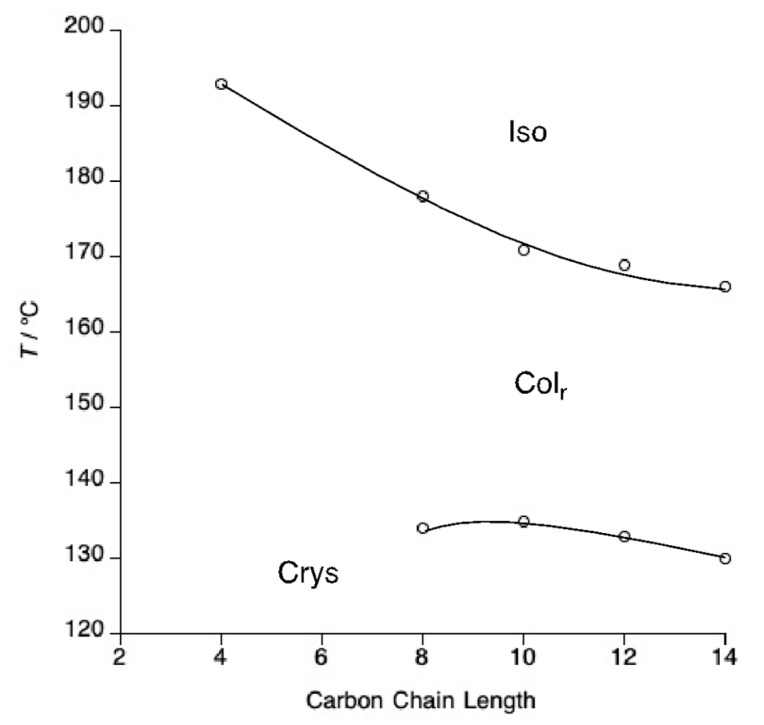
Structure and phase diagram of the tetracatenar triflimide salts, **5**-*n*.

**Figure 8:**
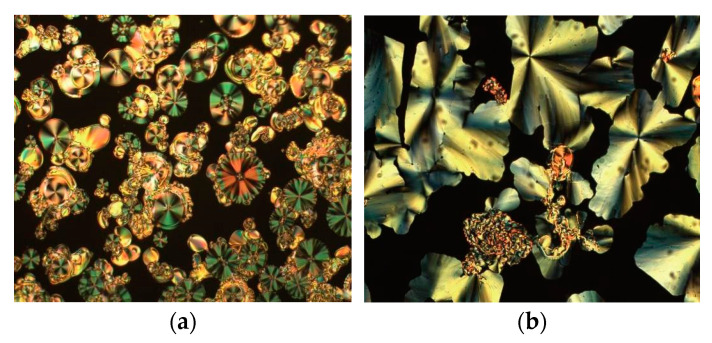

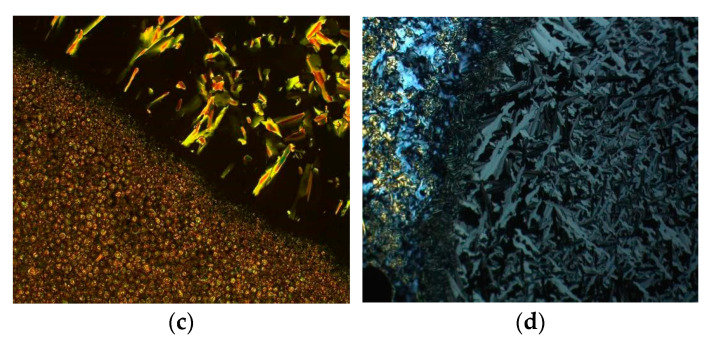
Photomicrographs of the Col_r_ phase formed by compound **5**-8: (**a**) cooling from the isotropic liquid at 10 °C min^−1^ at 158 °C, (**b**) slow cooling of the same compound at 0.1 °C min^−1^ at 159 °C, (**c**) miscibility gap between this phase and the Col_h_ phase of the OTf compound **1**-18 (**5**-8 is bottom left and **1**-18 is top right) and (**d**) miscibility gap between the phase of **5**-8 (top and left) and the SmA phase of the OTf compound **1**-12 (right).

The phase diagram is presented in Figure 7 (with the data collected in Table S8) and shows that all homologues from **5**-8 to **5**-14 were mesomorphic, while **5**-4 simply melted directly to an isotropic fluid. All compounds exhibited the same liquid crystal phase which presented a slightly odd, spherulitic texture that corresponded neither to a SmA nor a Col_h_ phase. In fact, only on cooling extremely slowly from the isotropic liquid (at 0.1 °C min^−1^) could any clear textures be obtained, which showed large pseudo focal-conic defects that would normally be associated with a columnar mesophase. Contact preparations between the triflimide salts and the SmA and Col_h_ phases of the triflate series showed a miscibility gap, so failing to provide any evidence as to the identity of this mesophase (see Figure 8c,d) for photomicrographs of these miscibility studies).

The melting points of the mesomorphic homologues were comparable to those of the triflate salts and remained fairly constant across the series (Table S9), while a small accompanying decrease in the clearing point was seen, so that the mesophase range reduced from 44 °C in **5**-8 to 36 °C in **5**-14. Compounds **5**-8 to **5**-14 melted into a mesophase with an odd spherulitic texture (Figure 8a) at normal cooling rate that corresponded neither to a SmA nor a Col_h_ phase. A clearer texture showing large, pseudo focal-conic defects typical of a columnar phase was obtained on much slower cooling from the isotropic liquid (Figure 8b). Contact preparations between the triflimide salt **5**-8 and the SmA phase of **1**-12 or with the Col_h_ phase of **1**-18 showed a miscibility gap as did a similar contact preparation with the SmA phase of a phenyl-1,2,4-triazolium triflimide [19], so failing to identity this mesophase (Figure 8c,d).

**Figure 9:**
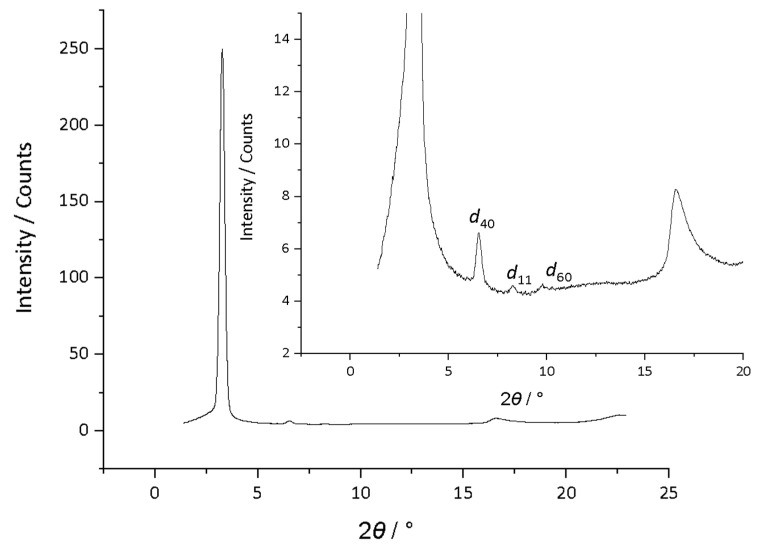
Diffraction pattern of compound **5**-10 at 139 °C: insert represents the zoomed in region for clarity of the low intensity reflections.

While optical microscopy could not identify the phase unequivocally, X-ray methods gave more insight and, on prolonged exposure, an additional medium-angle reflection could be observed (Figure 9), which could be indexed as the (11) reflection of a rectangular system, the low intensity likely reflecting weak correlations in this dimension. As the data show (Table 1), indexed in this was the *a*-dimension of the lattice increases with increasing terminal chain length (47.6 Å for **5**-8 to 65.6 Å for **5**-14), while the *b*-dimension remains effectively constant at 10.8 ± 0.1 Å. Unfortunately, the small number of reflections does not allow the plane group to be identified.

## 3. Discussion

**Figure 10:**
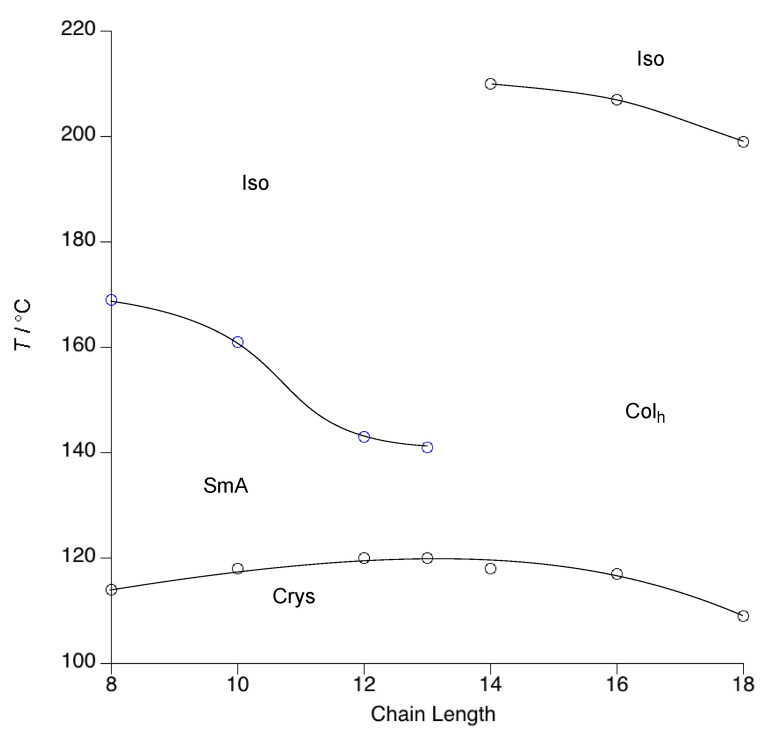
Phase diagram for **1**-*n* (reproduced from reference [15] with permission from the Royal Society of Chemistry).

The factors that control mesomorphism can be many and how they tension against one another from material to material can vary greatly. Therefore, to begin the discussion it is useful to recall the behaviour of the tetracatenar *N*-phenylpyridinium triflates (**1**-*n*) [15] (Figure 10) and first consider their behaviour in the light of that of neutral polycatenar liquid crystals. Thus, shorter-chain homologues of **1**-*n* show a SmA phase rather than the SmC phase seen in neutral analogues, which was rationalised by two factors. First were the strong electrostatic attractions between neighbouring anions and cations that stabilise self-organisation into layers and, indeed, the SmA phase is the most common (and often the only) mesophase seen for calamitic ionic mesogens. Second is that the effective molecular core volume is increased by the presence of the associated anion. This acts to cancel out the imbalance between core and chain cross-sectional areas at their interface, therefore removing the need for the mesogens to tilt in order to self-organise into layers (see the following references for a detailed discussion of driving forces in the mesomorphism in polycatenar liquid crystals [20,21]). These factors combine to allow the molecules to self-organise with their long axes orthogonal to the layer normal (the SmA phase) rather than having to tilt. It is then only at much longer chain lengths (*n* ≥ 14) that the lamellar phase can no longer be supported and a columnar phase is observed instead.

Although in cross-section the alkyl sulfate anions are reasonably similar to triflate, it is noticeable that, while in the triflate salts the Col_h_ phase (indicative of surface curvature) appears at *n* = 14, for octyl sulfate the lamellar phase is lost at *n* = 10 where a cubic phase is observed; for dodecyl sulfate, a cubic phase appears at *n* = 6. As such, there is a different or additional effect of the alkyl sulfate chains that affects the phase behaviour. In a detailed SAXS study of the mesomorphism of mono-alkoxy-stilbazole complexes of silver(I) with dodecyl sulfate anions for which a cubic phase is seen between a SmC and a SmA phase, the analysis led to the conclusion that the dodecyl chain extended alongside the cation and, importantly, beyond the rigid stilbazole core [22]. This conclusion was later demonstrated via the X-ray single crystal structure of related complexes that contained the dodecylene-1,12-disulfate anion [23]. The effect of this is that the anion chain contributes to the volume of the terminal chain and so leads to the disruption of lamellar phases at shorter terminal chain lengths on the cation. In fact, with the slightly shorter core of 4-phenylpyridine compared with stilbazole, this also occurs with octyl sulfate, too (length of the octyl sulfate anion is 11.3 Å [24], while half of the length of the phenylpyridinium cation is 8.4 Å [15]). Thus, both octyl sulfate and dodecyl sulfate anions promote the formation of a cubic phase in the *N*-phenylpyridinium salts in a manner analogous to that in the stilbazole complexes of silver(I) dodecyl sulfate. The longer dodecyl sulfate anion contributes even more to the terminal chain volume and so the cubic phase is seen at a shorter terminal chain length.

The other significant feature of note in these materials is the very significant mesophase stabilisation that is found within the homologous series when the columnar phase is introduced. Thus, for **3**-*n* the clearing point increases as 110 °C (*n* = 8, Cub-Iso), 159 °C (*n* = 10, Col_h_-Iso), 189 °C (*n* = 12, Col_h_-Iso), while for **4**-*n* the equivalent trend is 110 °C (*n* = 8, Cub-Iso), 159 °C (*n* = 10, Col_h_-Iso), 183 °C (*n* = 12, Col_h_-Iso). Recall also that for **1**-*n*, the clearing point jumped suddenly from 141 °C (*n* = 13, SmA-Iso) to 210 °C (*n* = 14, Col_h_-Iso) [15] and that this contrasts markedly with the behaviour of neutral, polycatenar materials (see Supplementary Information in reference 15). Indeed, revisiting the behaviour of the tetracatenar stilbazole complexes of silver(I), the same effect is seen even if the differences in clearing point are a little more modest [25].

As noted previously, lateral substituents have little effect on the phase diagrams of neutral, polycatenar compounds [26,27], although in reality the anions being considered here are very much bigger than the lateral substituents that are referenced. Nonetheless and as discussed, there is a strong tendency for rod-like ionic mesogens to form SmA phases, yet as well as stabilising the lamellar phase electrostatically, sterically they will act to destabilise it, and so there is a balance of two opposing factors. It was concluded therefore that on entering the columnar phase the destabilising effect of the anion is lost and the cation-anion pairs are more readily accommodated from a packing point of view, reflected in the phase stabilisation. As such, it is possible to conclude that the large difference in phase stability is due to the anion acting to destabilise the lamellar phases. Moreover, the fact that the same stabilisation is not found for the cubic phase when compared to the Col_h_ phase may suggest that the local organisation in the cubic phase is closer to that in the lamellar than in the columnar phase.

Now considering the triflimide salts **5**-*n*, the mesomorphism is different again and the X-ray data would appear to point to the formation of a mesophase of rectangular symmetry. Triflimide is much less flexible when compared to alkyl sulfates and somewhat more ‘board-shaped’ and much more charge-dispersed when compared to triflate. This last factor may be significant as the more dispersed electrostatic attraction may stabilise the SmA phase less well to allow the formation of the columnar phase, which in this case has rectangular plane symmetry. This is turn may result from the different nature of the packing of cation and anion on account of the shape of the anion, allowing the formation of the ribbon structure that characterises rectangular phases such as Col_r_, SmÃ and B_1_.

**Figure 11:**
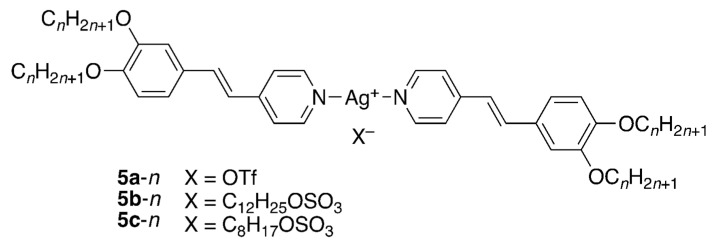
Structure of the silver complexes **5**-*n*.

It is then instructive to perform a second comparison of compounds **1**-*n*, **3**-*n* and **4**-*n* with related silver stilbazole complexes **5**-*n* (Figure 11) to which they bear significant structural similarity and whose phase diagrams are found in the Supplementary Information (Figures S11 and S12).

The first thing to note is that none of these complexes shows a lamellar phase and the phase diagrams show cubic and columnar organisation only. For the alkyl sulfate complexes, conductivity measurements showed that the complexes exist as tight-bound ion pairs and so in some ways they are more akin to neutral materials. For reasons discussed in detail elsewhere, [22] it is clear that there is intermolecular electrostatic attraction, but the absence of a lamellar phase suggests that it is likely to be weak, so that steric factors win out totally and a cubic phase is seen at shorter chain lengths. However, another significant factor is likely to be that, as evidenced by single-crystal X-ray structures for octyl sulfate [24] and triflate salts, [28] the complexes exist as dimers with the anions acting as a bridge between the two silver cations. Thus, in the phase diagram of **5b**-*n*, there is once more a significant phase stabilisation on introduction of the columnar phase, which is mirrored in **5c**-12 (the only homologue prepared for this anion), whereas the phase diagram for **5a**-*n* shows a much more modest stabilisation of the columnar phase. This observation would imply that the triflate is better ‘tolerated’ sterically in the dimeric arrangement within the cubic organisation which, as concluded above, is closer to that in the lamellar phase compared to the columnar phase.

## 4. Conclusions

The mesophase behaviour of these ionic, tetracatenar *N*-phenylpyridinium salts is remarkable for a variety of reasons. They represent extremely rare examples of ionic tetracatenar mesogens and, effectively, unique examples of tetracatenar mesogens showing a SmA phase, which behaviour is seen with triflate and alkyl sulfate anions. Perhaps the most intriguing feature of their phase behaviour is the observation that the columnar hexagonal phases of these salts are very much more stable than that of the SmA phases, with some very sharp increases in clearing point observed. It is argued that this in fact arises from a destabilisation of the lamellar phase consequent on the presence of the anion, which acts as a destabilising lateral group. That said, these effects are evidently subtle, for when the anion is changed to triflimide the ribbon-like Col_r_ phase is seen for all homologues studied, showing that in these salts the simple lamellar phase cannot be stabilised. This argument is supported further by comparisons with the silver stilbazole salts where reduced intermolecular electrostatic attractions precludes the formation of lamellar phases. Further, the better steric tolerance of the smaller triflate anion on account of the dimeric structure of the silver salts results in a smaller stabilisation on entering the columnar phase.

As such, these salts show a delicate interplay between electrostatic interactions, charge density and the steric effects of accommodating both anions in relation to their cations and terminal chains in relation to their cores. Therefore, the study of liquid crystals with multiple competing organisational requirements represents a fertile area for study rewarded by the possibility of unanticipated observations.

## Figures and Tables

**Table 1:** Diffraction data for series **5**-*n*.

*n*	*d*_obs_/Å	*hk*	Parameter/Å
8	23.8	20	*a* = 47.6
	11.8	40	*b* = 10.9
	10.6	11	
	8.8	60	
	5.3	halo	
10	27.0	20	*a* = 54.0
	13.5	40	*b* = 10.9
	10.7	11	
	9.0	60	
	5.3	halo	
12	29.9	20	*a* = 59.8
	15.0	40	*b* = 10.7
	10.5	11	
	10.0	60	
	5.3	halo	
14	32.8	20	*a* = 65.6
	16.4	40	*b* = 10.7
	10.6	11	
	5.3	halo	

## Data Availability

Raw data can be made available by the corresponding author.

## Supplementary Materials

The following are available online, Experimental details; thermal and X-ray data and a selection of figures.

## Sample Availability

Samples of some compounds may be available upon request to the corresponding author.
