# Supplementary material for: Liquid Crystals Based on the N-Phenylpyridinium Cation—Mesomorphism and the Effect of the Anion
Source: Molecules. 2021 May 1;26(9):2653. doi: 10.3390/molecules26092653 (PMC8124330; doi:10.3390/molecules26092653)
Supplement: Supplementary file 1 [file molecules-26-02653-s001.zip › molecules-1199297-supplementary.pdf]

Liquid Crystals based on the *N*-Phenylpyridinium Cation – Mesomorphism and the Effect of the Anion

Jordan D. Herod and Duncan W. Bruce*

Department of Chemistry
University of York
Heslington
YORK YO10 5DD (UK)
Tel: (+44) 1904 324085
E-mail: duncan.bruce@york.ac.uk

Supplementary Information

Nuclear Magnetic Resonance (NMR):

^{1}H, ^{19}F and $^{13}C\{^{1}H\}$ NMR spectra were recorded on a Jeol ECS400 spectrometer equipped with a sample changer operating at 400 MHz (^{1}H), 376 MHz (^{19}F) and 101 MHz (^{13}C), each at a temperature of 298 K.

Polarised Optical Microscopy (POM):

Liquid crystal textures were observed using an Olympus BX50 Optical Microscope equipped with a Linkam Scientific LTS350 heating stage, Linkam LNP2 cooling pump and a Linkam TMS92 controller.

Differential Scanning Calorimetry (DSC):

Calorimetry scans were run on a Mettler Toledo DSC822e, (running on a Stare software) equipped with a TSO801R0 sample robot and calibrated using pure indium. Samples were run at heating/cooling rates of 5 °C min^{-1}. DSC data mentioned in the article are onset temperatures.

Elemental Analysis:

Analysis was carried out on an Exeter Analytical Inc CE 440 Elemental Analyser.

Small-Angle X-ray Scattering (SAXS):

Small angle X-ray scattering was performed using a Bruker D8 Discover equipped with a temperature-controlled, bored-graphite rod furnace, custom built at the University of York. The radiation used was CuKα (λ = 0.154056 nm) from a 1 µS microfocus source. Diffraction patterns were recorded on a 2048 x 2048 pixel Bruker VANTEC 500 area detector.

Synthetic Procedures:

Preparation of the tetracatenar triflimide salts, **5**-*n*

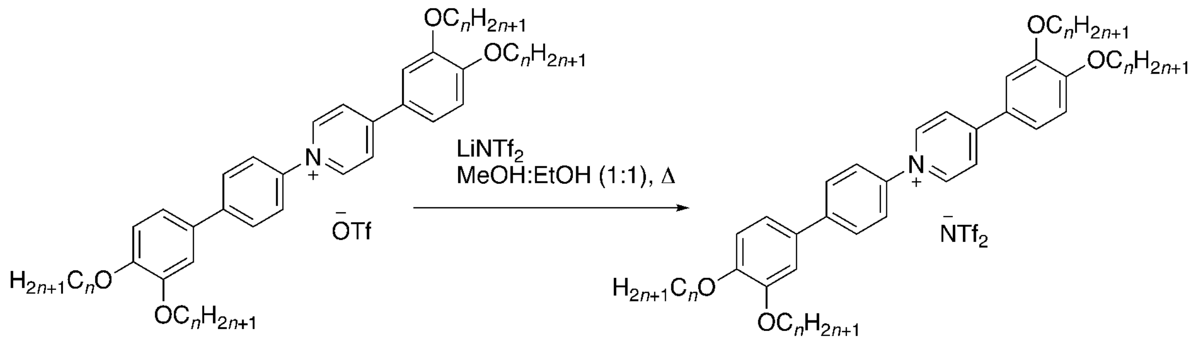

1-*n* **5**-*n*

Figure S1. Metathesis of triflate anion for triflimide.

Triflate salt **1**-*8* (0.05 g, 0.052 mmol) was taken into ethanol: methanol (1:1, 50 ml) and heated to 70 °C. $LiNTf_2$ (0.037 g, 0.13 mmol) was then added in methanol (5 ml) and the reaction mixture stirred for 2 h. Water was then added dropwise to precipitate the product as its triflimide salt, which was subsequently isolated *via* filtration and washed multiple times with water. The product was then purified by flash column chromatography on silica gel using dichloromethane: methanol as the eluent (95:5) to afford a bright orange solid on evaporation of the solvent (76%): ^{1}H NMR (400 MHz, $CDCl_3$) δ 8.76 (2H, AA'XX', *J* = 7.0 Hz), 8.30 (2H, AA'XX', *J* = 7.0 Hz), 7.80 (2H, AA'XX', *J* = 8.5 Hz), 7.68 (2H, AA'XX', *J* = 8.5 Hz), 7.52 (1H, dd, *J* = 8.5, 2.5 Hz), 7.42 (1H, d, *J* = 2.5 Hz), 7.14 (2H, m), 7.01 (1H, d, *J* = 8.5 Hz), 6.97 (1H, d, *J* = 8.5 Hz), 4.11 (2H, t, *J* = 6.5 Hz), 4.08 (2H, t, *J* = 6.5 Hz), 4.07 (2H, t, *J* = 6.5 Hz), 4.04 (2H, t, *J* = 6.5 Hz), 1.85 (8H, m), 1.49 (8H, m), 1.32 (32 H, m), 0.88 (12H, m). ^{19}F NMR (376 MHz, $CDCl_3$) δ -78.54 (s).

Table S1. Micro-analytical data for the tetracatenar triflimide salts **5**-*n*: theoretical values in parentheses.

n	Yield / %	%C	%H	%N
4	64	56.2 (56.2)	5.7 (5.8)	2.9 (3.2)
8	76	62.2 (62.2)	7.5 (7.5)	2.4 (2.5)
10	81	64.6 (64.3)	8.3 (8.1)	2.2 (2.3)
12	69	66.2 (66.1)	8.7 (8.7)	2.1 (2.1)
14	83	67.6 (67.7)	9.3 (9.1)	1.8 (2.0)

Preparation of chloride salts, **2**-*n*

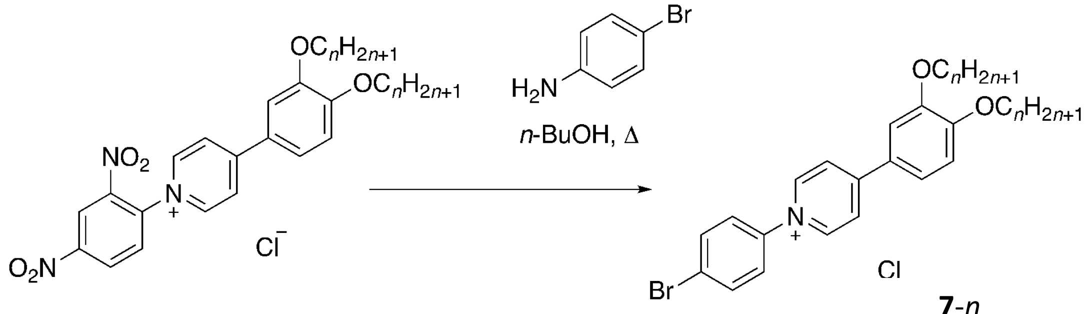

6-*n*

7-*n*

Figure S2. Preparation of chloride salts **7**-*n.*

2,4-Dinitro salt **6**-*8* (2.79 g, 4.55 mmol) was taken into *n*-butanol and heated to 110 °C under a flow of nitrogen. 4-Bromoaniline (3.12 g, 18.2 mmol) was then added and the reaction mixture stirred for 16 h before being cooled to room temperature and the precipitate isolated *via* filtration. The resulting orange solid was washed multiple times with acetone and then triturated with dichloromethane to afford a bright yellow solid (45%): ^{1}H NMR (400 MHz, $CDCl_3$) δ 8.94 (2H, AA'XX', *J* = 7.5 Hz), 8.32 (2H, AA'XX', *J* = 7.5 Hz), 7.76 (2H, AA'XX', *J* = 8.5 Hz), 7.67 (2H, AA'XX', *J* = 8.5 Hz), 7.57 (1H, dd, *J* = 8.5, 2.5 Hz), 7.40 (1H, d, *J* = 2.5 Hz), 6.98 (1H, d, *J* = 8.5 Hz), 4.06 (2H, t, *J* = 6.5 Hz), 4.04 (2H, t, *J* = 6.5 Hz), 1.80 (4H, m), 1.43 (4H, m), 1.26 (16H, m), 0.82 (3H, t, *J* = 7.0 Hz), 0.81 (3H, t, *J* = 7.0 Hz).

Preparation of tetracatenar chloride salt **5**-*n*

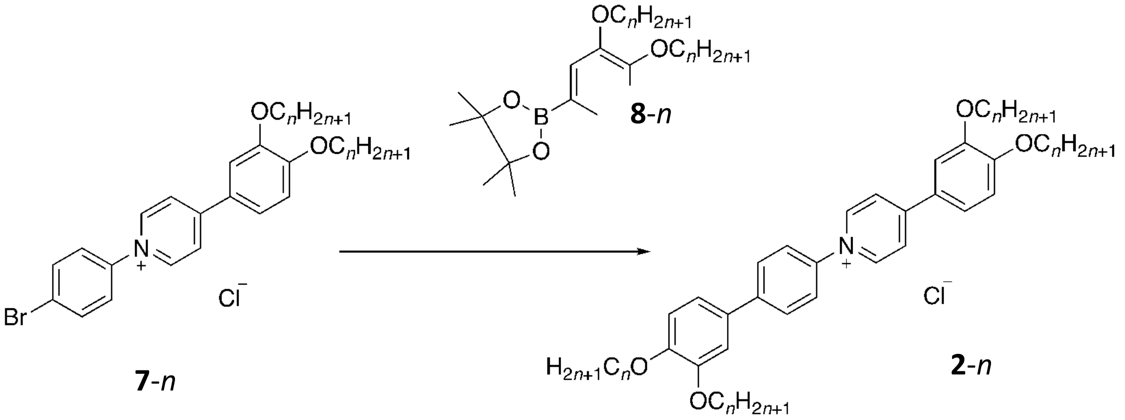

Figure S3. Preparation of the tetracatenar chloride salts **2**-*n*, used as intermediates in the synthesis of the alkyl sulfates.

Salt **7**-*8* (0.05 g, 0.076 mmol) and 3,4-dialkoxybenzeneboronic acid pinacol ester **8**-*8* (0.047 g, 0.09 mmol) were added to a three-necked round-bottomed flask already purged with nitrogen and taken into THF (50 ml) and ethanol (25 ml). Aqueous sodium carbonate (50 ml, 2 mol dm^{-3}) was then added and the biphasic solution degassed with arogon whilst undergoing agitation in an ultrasonic bath. [$Pd_3(OAc)_6$] (3.5 x 10^{-3} g, 1.52 x 10^{-3} mmol) and SPhos (7.0 x 10^{-3} g, 3.0 x 10^{-3} mmol) were dissolved in anhydrous THF (1 ml) and stirred under nitrogen until the mixture became red. This pre-prepared catalyst was then added to the reaction mixture, which was then heated to 75 °C under a steady flow of nitrogen. After complete consumption of the limiting reagent (after 6 h), the reaction mixture was cooled to r.t. and the biphasic mixture separated. The aqueous layer was extracted with dichloromethane (3 x 50 ml) and the combined organic extracts were washed with aqueous NaCl (100 ml, saturated) and dried over anhydrous $MgSO_4$ before evaporation of the solvent under reduced pressure. The product was purified by flash column chromatography on silica gel using dichloromethane:methanol (95:5) as the eluent. On evaporation of the solvent, the solid was finally triturated with dichloromethane (50 ml) to afford a bright orange solid (86%): ^{1}H NMR (400 MHz, $CDCl_3$) δ 9.37 (2H, AA'XX', *J* = 7.0 Hz), 8.40 (2H, AA'XX', *J* = 7.0 Hz), 7.92 (2H, AA'XX', *J* = 9.0 Hz), 7.76 (2H, AA'XX', *J* = 9.0 Hz), 7.53 (1H, dd, *J* = 8.5,

2.5 Hz), 7.36 (1H, d, *J* = 2.5 Hz), 7.09 (2H, m), 6.94 (1H, d, *J* = 8.5 Hz), 6.92 (1H, d, *J* = 8.5 Hz), 4.09 (2H, t, *J* = 6.5 Hz), 4.04 (6H, m), 1.84 (8H, m), 1.49 (8H, m), 1.31 (32H, m), 0.87 (12H, m).

Preparation of the tetracatenar octyl sulfate salts, **3**-*n*

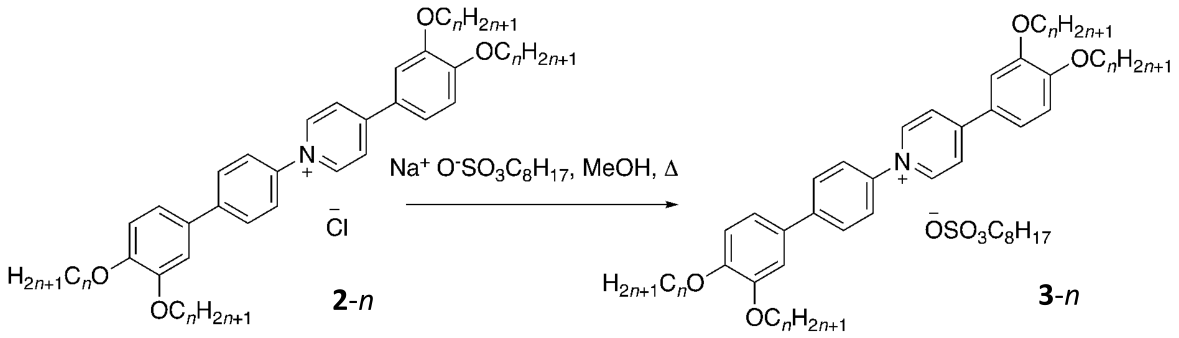

Figure S4. Preparation of the octyl sulfate salts, **3**-*n*, from the chloride precursors.

The following procedure documents the preparation of **3**-8: All other derivatives were prepared in an analogous fashion, except that ethanol was used as the reaction solvent for the *n* = 12 homologue due to the insolubility of this compound in hot methanol. The tetradecyloxy derivative was, however, prepared successfully in an identical fashion from the triflate salt using ethanol as the reaction solvent.

Chloride salt **2**-*8* (0.03 g, 0.035 mmol) was taken into methanol (75 ml) and the solution heated to 60 °C. The solution was then saturated with sodium octyl sulfate and stirred at this temperature for 2 h. Water was then added dropwise to precipitate the product as its octyl sulfate salt, which was subsequently isolated *via* filtration and washed with multiple portions of water. The compound was then crystallised from hot ethanol and the solid taken into dichloromethane and filtered through a 0.2 µm PTFE filter to remove any insoluble impurities. The solvent was then removed and the compound dried under vacuum (66%): ^{1}H NMR (400 MHz, $CDCl_3$) δ 8.98 (2H, AA'XX', *J* = 7.0 Hz), 8.41 (2H, AA'XX', *J* = 7.0 Hz), 7.72 (2H, AA'XX', *J* = 8.5 Hz), 7.67 (2H, AA'XX', *J* = 8.5 Hz), 7.49 (1H, dd, *J* = 8.5, 2.5 Hz), 7.38 (1H, d, *J* = 2.5 Hz), 7.05 (1H, d, *J* = 2.5 Hz), 7.03 (1H, dd, *J* = 8.5, 2.5 Hz), 6.88 (1H, d, *J* = 8.5 Hz), 6.84 (1H, d, *J* = 8.5 Hz), 4.11 (4H, m), 4.04 (2H, t, *J* = 6.5 Hz),

4.01 (2H, t, J = 6.5 Hz), 3.94 (2H, t, J = 7.0 Hz), 1.84 (8H, m), 1.69 (2H, m), 1.29 (42 H, m), 0.87 (12 H, m), 0.83 (3H, t, J = 7.0 Hz).

Table S2. Micro-analytical data for the tetracatenar octyl sulfate salts **3**-*n*: theoretical values in parentheses.

n	Yield / %	%C	%H	%N
6	82	71.3 (71.9)	9.3 (9.1)	1.5 (1.5)
8	66	73.5 (73.4)	10.1 (9.7)	1.6(1.4)
10	71	74.3 (74.6)	10.2 (10.1)	1.1 (1.2)
12	74	75.6 (75.2)	10.4 (10.5)	1.0 (1.1)
14	68	76.1 (76.4)	11.3 (10.8)	1.0 (1.0)

Preparation of the tetracatenar dodecyl sulfate salts, **4**-*n*

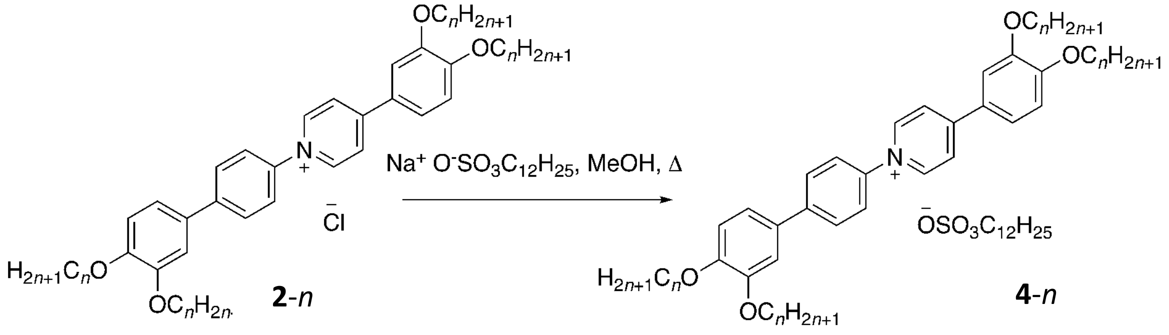

Figure S5. Preparation of the dodecyl sulfate salts, **4**-*n*, from the chloride precursors.

The following procedure documents the preparation of the **4**-8, all other derivatives were prepared in an analogous fashion except that ethanol was used as the reaction solvent for the *n* = 12 derivative due to the insolubility of this compound in hot methanol.

Chloride salt **5**-*8* (0.03 g, 0.035 mmol) was taken into methanol (75 ml) and heated to 60 °C. This solution was then saturated with sodium dodecyl sulfate and the reaction mixture stirred at this temperature for 2 h. Water was then added dropwise to precipitate the product as its dodecyl sulfate salt, which was subsequently isolated *via* filtration and the solid washed multiple times

with water. The product was then crystallised from hot ethanol and the resulting solid taken into dichloromethane and filtered through a 0.2 μm PTFE filter to remove any insoluble impurities. The solvent was then removed and the product dried under vacuum (65%): ^{1}H NMR (400 MHz, $CDCl_3$) δ 8.99 (2H, AA'XX', *J* = 6.5 Hz), 8.39 (2H, AA'XX', *J* = 6.5 Hz), 7.73 (2H, AA'XX', *J* = 8.5 Hz), 7.68 (2H, AA'XX', *J* = 8.5 Hz), 7.49 (1H, dd, *J* = 8.5, 2.5 Hz), 7.39 (1H, d, *J* = 2.5 Hz), 7.05 (2H, m), 6.89 (1H, d, *J* = 8.5 Hz), 6.86 (1H, d, *J* = 8.5 Hz), 4.11 (4H, t, *J* = 7.0 Hz), 4.04 (2H, t, *J* = 7.0 Hz), 4.01 (2H, t, *J* = 7.0 Hz), 3.96 (2H, t, *J* = 7.0 Hz), 1.84 (8H, m), 1.69 (2H, m), 1.49 (8H, m), 1.30 (50H, m), 0.87 (15H, m).

Table S3. Micro-analytical data for the tetracatenar dodecyl sulfate salts **4**-*n*: theoretical values in parentheses.

n	Yield / %	%C	%H	%N
6	78	72.1 (72.7)	8.9 (9.4)	0.9 (1.4)
8	65	73.8 (74.1)	10.0 (9.90)	0.7 (1.3)
10	69	75.1 (74.7)	10.3 (10.4)	1.2 (1.3)
12	73	75.4 (76.0)	10.6 (10.7)	1.4 (1.1)

Thermal data:

Table S4. Thermal data for the octyl sulfate salts, **3**-*n,* from microscopy: *marks a transition not detected *via* DSC.

n	Transition	*T* / °C	Δ*H* / kJ mol^{-1}
6	Crys-Crys'	54	0.9
	Crys'-SmA	93	42.9
	SmA-Iso	130	0.8
8	Crys-Crys'	53	11.6
	Crys'-SmA	81	40.5
	SmA-Iso	101	1.8
10	Crys-SmA	89	65
	SmA-Cub	96	*
	Cub-Iso	121	1.5
12	Crys-SmA	89	35.2
	SmA-Cub	94	*
	Cub-Col$_h$	106	*
	Col$_h$-Iso	183	*
14	Crys-Col$_h$	83	33.5
	Col$_h$-Iso	189	*

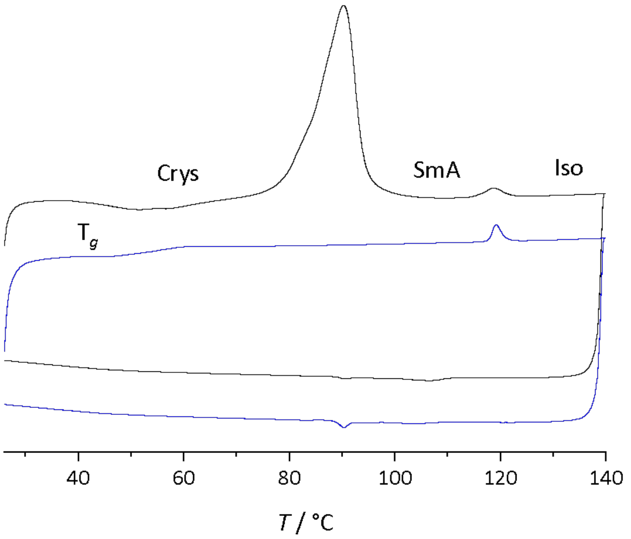

Figure S6. DSC of compound **3**-*10* showing glass transition, T_g, on second heat (blue curve).

Table S5. Diffraction data for the octyl sulfate salts, **3**-*n*.

n	**d_{obs} / Å**	***hk(l)***	***Mesophase***
6	26.4	*001*	SmA
	13.2	*002*	
	4.7	halo	
8	28.7	*001*	SmA
	4.7	halo	
10	30.5	-	Cub
	26.6	-	
	4.7	halo	
12	30.1	*10*	Col_h
	-	*11*	
	4.7	halo	
14	33.1	*10*	Col_h
	19.1	*11*	
	4.7	halo	

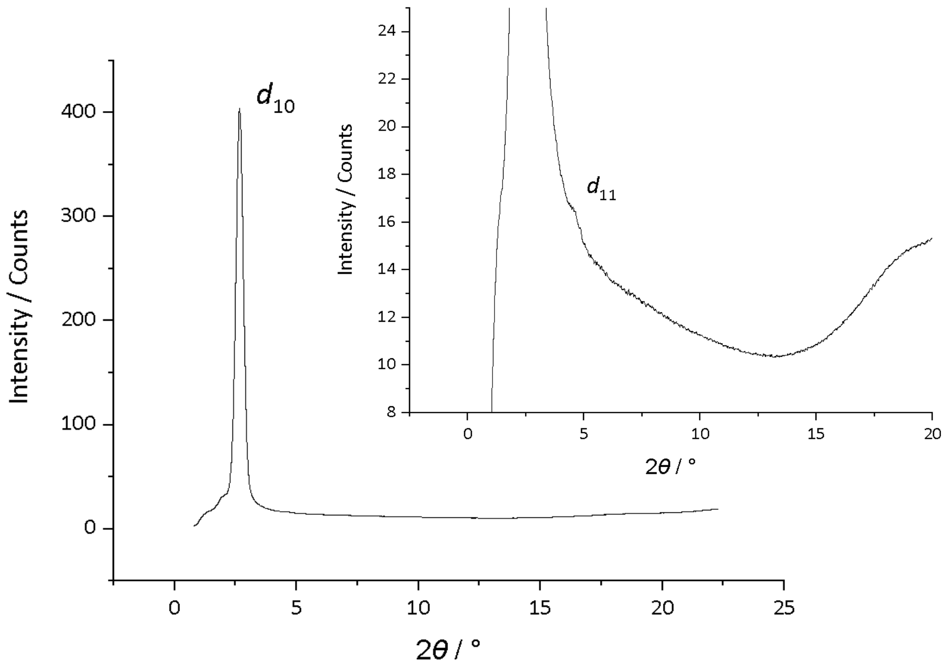

Figure S7. Diffraction pattern of the hexagonal columnar phase formed by compound **3**-*14* at 116 °C: inset shows the weak d_{11} reflection.

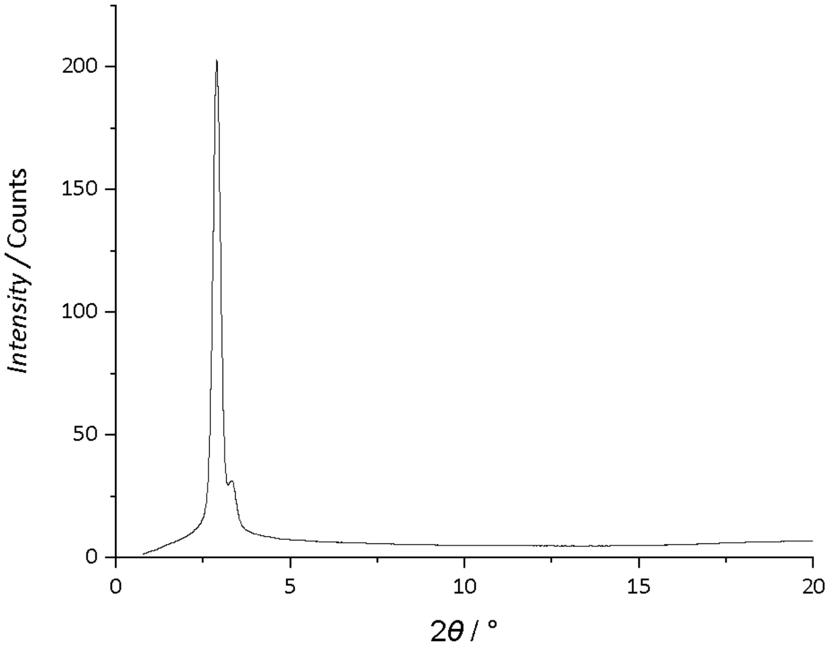

Figure S8. Diffraction pattern of the cubic phase formed by **3**-*10*.

Table S6. Thermal data for the dodecyl sulfate salts, **4**-*n*: *marks a transition not detected *via* DSC.

n	Transition	*T* / °C	Δ*H* / kJ mol^{-1}
6	Crys-SmA	93	42.6
	SmA-Cub	95	*
	Cub-SmA	115	0.8
	SmA-Iso	133	0.7
8	Crys-SmA	83	32.5
	SmA-Cub	101	*
	Cub-Iso	110	1.1
10	Crys-Cub	95	58
	Cub-Col_h	121	0.8
	Col_h-Iso	159	0.5
12	Crys-Col_h	96	87.9
	Col_h-Iso	189	1.7

Table S7. Diffraction data for the dodecyl sulfate salts, **4**-*n*.

n	***d_{obs}* / Å**	***hk(l)***	**Mesophase**
6	26.8	*001*	SmA
	4.7	halo	
8	28.8	*001*	SmA
	4.7	halo	
8	34.0	-	Cub
	25.8	-	
	4.7	halo	
10	30.2	-	Cub
	26.4	-	
	4.7	halo	
10	31.1	*10*	Col_h
	17.9	*11*	
	15.5	*20*	
	4.7	halo	
12	32.1	*10*	Col_h
	-	*11*	
	16.0	*20*	
	4.7	halo	

Table S8. Thermal data for the triflimide salts, **5**-*n*.

n	Transition	*T* / °C	Δ*H* / kJ mol^{-1}
4	Crys-Iso	188	6.4
	Crys'-Iso	193	5.3
8	Crys – Col_r	134	10.9
	Col_r - Iso	178	5.7
10	Crys - Crys'	70	16.1
	Crys'- Col_r	135	15.3
	Col_r - Iso	171	7.8
12	Crys - Crys'	79	49.7
	Crys'- Col_r	133	34.2
	Col_r - Iso	169	21.6
14	Crys-Crys'	88	78.5
	Crys'- Col_r	130	52.1
	Col_r - Iso	166	29.8

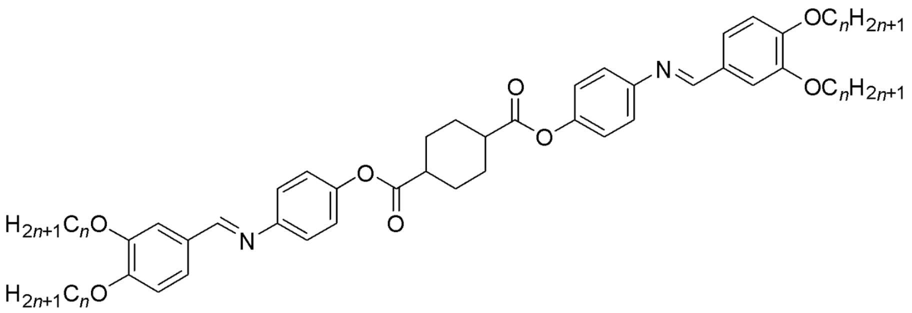

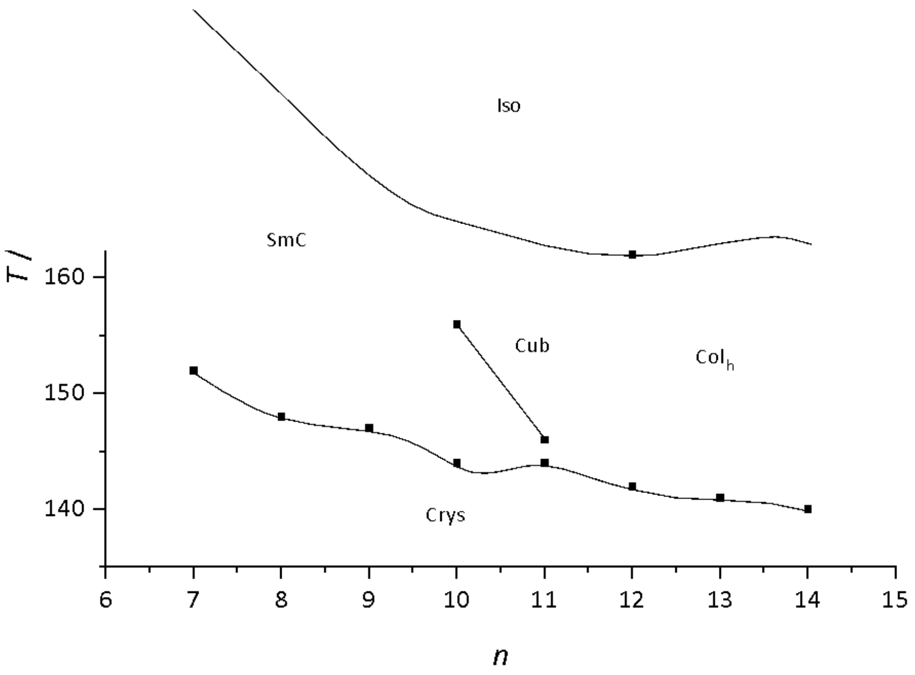

Figure S9. Phase diagram of a neutral tetracatenar liquid crystal reproduced from: C. Alstermark *et al.*, *Liq. Cryst.*, 1990, **8**, 75–80.

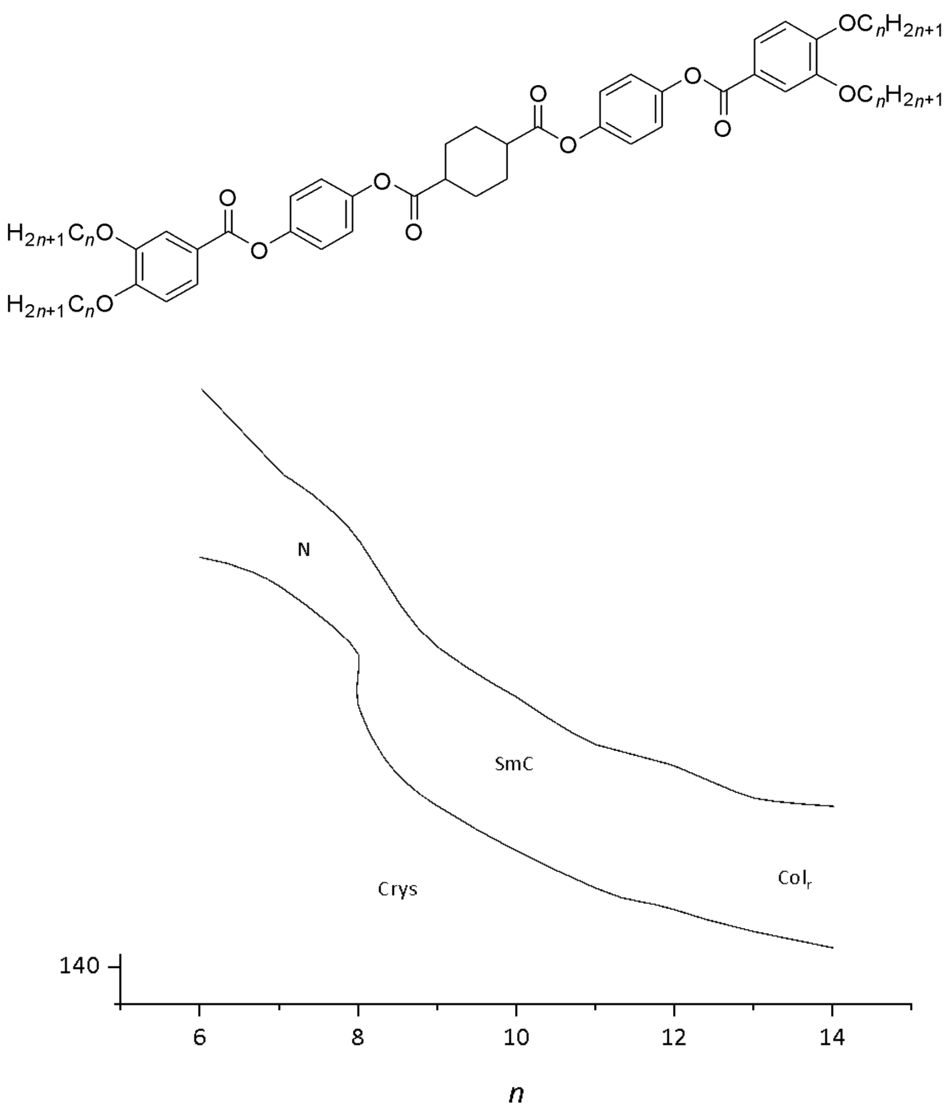

Figure S10. Phase diagram of a second neutral tetracatenar liquid crystal reproduced from: H. T. Nguyen *et al.*, *Liq. Cryst.*, 1990, **8**, 797–811.

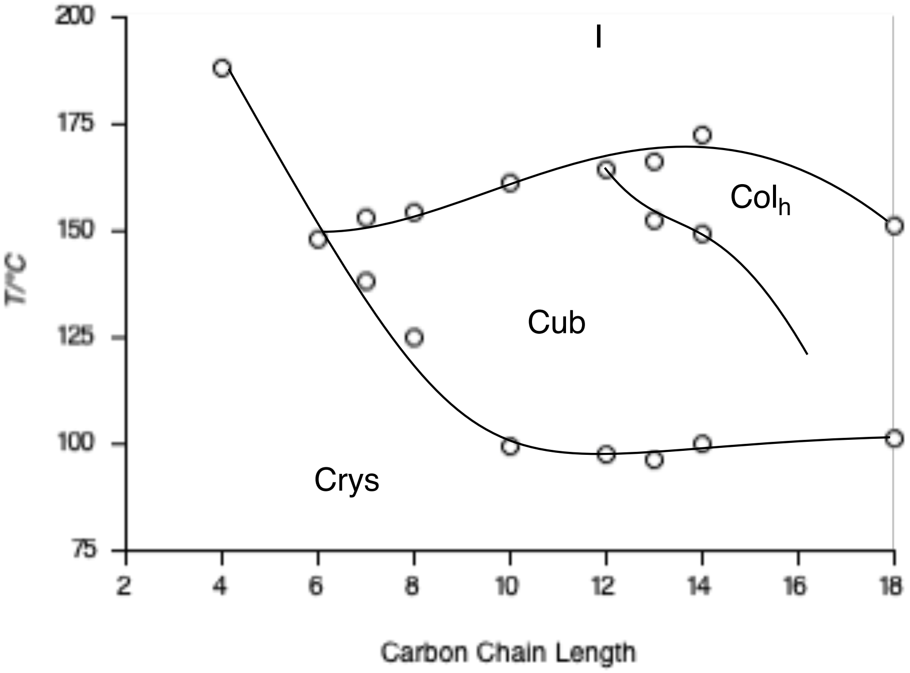

Figure S11 Phase diagram for *bis*(3,4-dialkoxystilbazole)silver(I) dodecyl sulfates. Reproduced from Bruce, D. W. *Acc. Chem. Res.*, 2000, **33**, 831-40 courtesy of the American Chemical Society.

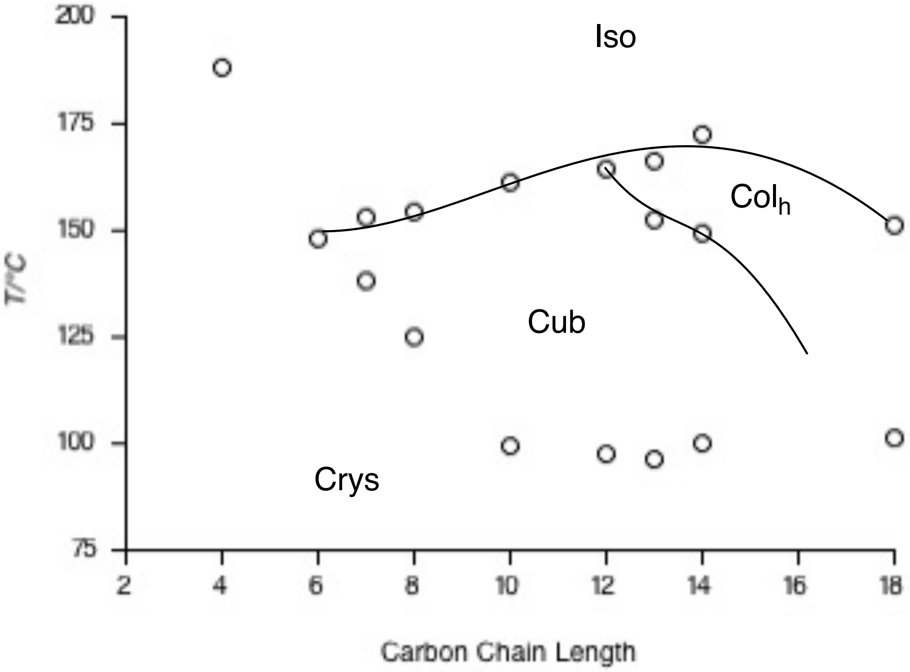

Figure S12 Phase diagram for *bis*(3,4-dialkoxystilbazole)silver(I) triflates. Reproduced from Bruce, D. W. *Acc. Chem. Res.*, 2000, **33**, 831-40 courtesy of the American Chemical Society.
